# The novel testicular enrichment protein Cfap58 is required for Notch-associated ciliogenesis

**DOI:** 10.1042/BSR20192666

**Published:** 2020-01-17

**Authors:** Zheng-Zheng Li, Wen-Long Zhao, Gui-Shuan Wang, Ni-Hao Gu, Fei Sun

**Affiliations:** 1 International Peace Maternity & Child Health Hospital Affiliated to Shanghai Jiao Tong University School of Medicine, Shanghai 200030, China; 2 Shanghai Key Laboratory for Reproductive Medicine, School of Medicine, Institute of Embryo-Feta Original Adult Disease, Shanghai Jiao Tong University, Shanghai 200030, China; 3 Institute of Reproductive Medicine, School of Medicine, Nantong University, Nantong 226001, Jiangsu, China

**Keywords:** centrosomes, cilia, notch signalling pathway

## Abstract

Cilia and flagella are critical organelles with conserved internal structures and diverse developmental and physiological processes according to cell type. Although the core components of structures are shared with thousands of associated proteins involved in cilia or flagella formation, we hypothesized that some unknown proteins, such as outer dense fiber 2 (Odf2/Cenexin) perform distinct functions in these organelles. In the present study, we identified several uncharacterized proteins through mass spectrometry interactome analysis of Odf2/Cenexin proteins. We further examined the expression patterns and functions of a protein named cilia and flagella associated protein 58 (Cfap58) in cultured astrocytes and sperm flagella. The results of a combination of biochemical analyses and drug administration studies reveal that Cfap58 is a testis-enrichment protein that exhibits similar localization to Odf2/Cenexin proteins and is required for the elongation of the primary cilium and sperm midpiece via modulation of the Notch signaling pathway. However, the cell cycle-related functions and localization of Odf2/Cenexin in the mother centriole were not altered in Cfap58 knockdown cells. These findings indicate that Cfap58 may be partially recruited by Odf2/Cenexin proteins and is indispensable for the cilia and flagellar assembly. These data provide us with a better understanding of ciliogenesis and flagellar elongation and may aid in identifying new targets for diseases caused by Notch-mediated ciliopathies and flagellar abnormalities.

## Introduction

Flagella and cilia are ancient and evolutionarily conserved organelles that are microtubule-based structures emanating from basal bodies [1,2]. In mammals, the cilia are widely distributed in various cell types and are generally categorized as motile cilia and sensory, non-motile cilia, also known as primary cilia [3]. Motile cilia are generally found in the epithelium of the conducting airways, oviducts, efferent ductules and cochlea, and function in mucociliary clearance, oocyte/embryo/sperm transportation and ventricular fluid flow, respectively [4–9]. On the other hand, primary cilia are deemed to be antenna that interpret a number of morphogens, such as Sonic Hedgehog (Shh) protein, bone morphgenetic protein (BMP) and Wnt and Notch proteins [10–13]. The impairments in primary cilia in function and structure have manifested a broad spectrum of diseases overall known as ‘Ciliopathy’ in humans, including polycystic kidney disease (PKD), Bardet–Biedl syndrome (BBS) and other developmental defects [14].

Similar to cilia, the flagella are nucleated from basal body to establish longer 9+2 microtubule arrangements in the center to form axonemes [15]. In general, the axonemes of sperm flagella are surrounded by nine precise structures, called outer dense fibers (ODFs), to execute the function of a propulsive engine in mammalian sperm [16]. Dysfunctions of flagella are major causes of male infertility or subinfertility [16,17].

Due to the similarity of internal structures between cilia and flagella, it is believed that the protein components of cilia and flagella for structural foundation are the same [18]. Therefore, extensive studies have focused on providing information about the common protein composition of cilia and flagella through comparative genomics and transcriptomics in cell lines and different species. For instance, it has been demonstrated that some of the BBS and the intraflagellar transport (IFT) family proteins play critical roles in both cilia and flagella [19–21]. Although some excellent studies have revealed that several distinct proteins are recruited by cilia and sperm flagella, the majority of results definitely certificate the idea that the enormously conserved proteins are involved in the structures and functions both of cilia and flagella [22]. Nevertheless, it is notable that some of the common proteins exhibit distinct localizations and functions in cilia and flagellum. For example, outer dense fiber 2 (Odf2/Cenexin) proteins were initially found in ODFs that are required for axoneme stability and sperm motility; later, it was determined that they were localized on the appendage of mother centrioles in somatic cells [23,24]. Disruption of Odf2/Cenexin expression in ciliated cells led to ciliogenesis deficiency [25]. Thus, identifying more of these proteins may help us gain a better understanding of the similarities and differences between cilia and flagellar assembly.

In the present study, we applied mass spectrometry interactome analysis of the Odf2/Cenexin proteins in sperms to find novel proteins involved in ciliogenesis and flagellar formation (Supplementary Table S1). On validation, we found that cilia and flagella associated protein 58 (Cfap58) is a novel protein expressed abundantly in sperms and ciliated cells. Reduced expression level of Cfap58 in astrocytes resulted in the impairment of primary cilia growth without affecting cell cycle progression and the capacity of the microtubule organization center (MTOC). Inhibition of Notch signaling elevated not only the expression level of Cfap58 *in vitro* and *in vivo*, but also the length of the primary cilia and sperm midpiece.

## Materials and methods

### Animal care and use

The wild-type C57BL/6J mice were purchased from the SLAC Laboratory Animal Co., Ltd. (Shanghai, China) and maintained under specific-pathogen-free (SPF) conditions for sample collection and drug administration assay. The experiments on animals followed the guidelines of the Animal Care and Use Committee of the Shanghai Jiao Tong University, School of Medicine. The mice were anesthetized in a chamber containing 2% isoflurane mixed with 0.2 l/min 100% O_2_ and maintained with a face mask (0.5% isoflurane). And then, the mice were killed following the CO_2_ approach after tissue collection. The animal experiments took place at Shanghai Jiao Tong University, School of Medicine, and approved by the Jiao Tong University School of Medicine Animal Ethics Committee (approval number GKLW2016-31).

### Cell culture, treatment, transfection and viral generation and infection

HEK293T cells were plated on 10-cm dishes and cultured in DMEM supplemented with 10% fetal bovine serum (FBS). HA-Cfap58 plasmid combined with the GFP vector, Odf2-GFP vector or Cenexin-GFP vector, respectively, were transfected into the cells using Lipofectamine 2000 for the pulldown assay. For the RNA interference (RNAi) efficiency assay, the HEK293T cells were transfected with HA-Cfap58 and shRNA vectors for 72 h.

The mice astrocyte cells were dissociated from P14 mice midbrain and cultured as previously described [26]. Briefly, astrocyte cells were cultured in proliferation medium containing DMEM supplemented with 10% FBS, B27, 10 ng/ml epidermal growth factor (EGF) and 10 ng/ml fibroblast growth factors (FGF2) or in primary cilia-induced medium (proliferation medium with FBS withdrawal). For Notch signaling inhibition, LY411575 was dissolved in DMSO and added to induced medium at a final 4 or 6 mM for 48 h as previously described [27]. To examine the function of Cfap58 in ciliogenesis, astrocytes as models of ciliated cells were infected with the Cfap58 shRNA lentiviruses provided by the Ohio Company (Shanghai China) at a 10 multiplicity of infection (MOI) for 72 h.

### Plasmid construction and qPCR

Total RNA was extracted from tissues with TRIzol (15596026, Invitrogen, Carlsbad, CA, U.S.A.) and converted to cDNA using the PrimeScript™ II First Strand cDNA Synthesis Kit (No. 6210A, Takara, Japan). The Cfap58 cDNA fragment was amplified from adult testis cDNA library and cloned into a pcDNA3.0 empty vector digested by EcoR V and Xho I. For the qPCR assay, the samples were conducted with PrimeScript™ RT reagent with gDNA Erase (No. RR047Q, Takara, Japan) as previously described [28]. The RNAi-resistant Cfap58 against Cfap58 sh3 was performed by PCR-based mutagenesis. The mutant allele contains 5′-gAatgtcCatgaaTaaTatTt-3′ (from nt2522 to nt2542 in CDS) (mutant alleles are indicated in uppercase). The primers used in this section are listed in Supplementary Table S2.

The shRNA sequences targeted mouse Cfap58 were designed using an online design tool in Thermo Fisher Scientific and cloned into a pLKD vector under the control of the U6 promoter. The sequences were given as following Supplementary Table S2.

### Western blotting

First, the proteins from cells, mouse testes and sperms were prepared as previously described [28]. Next, the samples were loaded into SDS/PAGE gels to be performed using the enhanced chemiluminescence (ECL) detection system (Pierce, Rockford, IL, U.S.A.). All antibodies used in the present study are listed in Supplementary Table S3.

### Immunoprecipitation

After 48 h of transfection of the related plasmids, the HEK293T cells were lysed using a high salt lysis buffer as previously described [16]. The insoluble components were discarded via centrifugation, and the supernatant was incubated with GFP-linked beads at 4°C for 4 h. Next, the immunoprecipitates were washed three times with 1 ml of wash buffer and eluted with 50 μl of 0.5 M glycine buffer (pH 2.5) according to a previous study [16]. The elution solution was neutralized with 10 μl of Tris/HCl buffer (pH 8.0), and then resuspended in SDS loading buffer for Western blotting analysis.

### Indirect immunofluorescence microscopy and quantification

The sperms and the cells that were grown on a coverslip coated with or without PDL were fixed in 4% paraformaldehyde (PFA) for 10 min at room temperature for cilia immunostaining or in methanol for 10 min at −20°C for immunostaining of the centrosome and flagellar proteins. And then the sperms and cells were washed three times with PBS quickly at room temperature following standard immunofluorescence procedures [29]. The primary and secondary antibodies were listed in Supplementary Table S3. The nuclei were stained by Hoechst34580 (63493, Sigma, U.S.A.) at room temperature for 30 min. The fluorescence images were obtained at 0.5–1 μm intervals in z-axis with SP8 confocal microscope (Leica Microsystems, Wetzlar, Germany). The percentage and length of cilia per cell as well as the sizes and mean α-tubulin intensities of asters were measured using ImageJ software as previously described [30,31].

### Sperm preparation

The caudal epididymis was removed from the anesthetic adult mice treated with or without LY411575 for 35 days and cut by eye scissors into small bulks. Next, these tissues were submerged in 500 μl of Tyrode’s salt solution (T2397, Sigma, U.S.A.) at 5% CO_2_, 37°C incubator for 15 min. The sperms swam out into the salt solution after incubation, and were obtained by centrifugation at 600 ***g*** for 5 min at room temperature. Next, the sediments were re-suspended with 1 ml of PBS. Two aliquots of suspension (each 10 μl) were taken out to perform with computer-assisted sperm analysis software (CASA software) as previously described and Giemsa staining (G1020, Solarbio, China) was performed according to the production manual [16]. The length of the sperm midpiece was measured using ImageJ software.

### Cell cycle assay

The cell cycle analysis was performed using flow cytometry (FCM). Cells were seeded on to a six–well plate and infected by Cfap58 shRNA3 or scramble lentivirus. At 72 h after infection, cells were collected and fixed with 70% ice ethanol overnight at 4°C. The centrifuged cells were subsequently stained with propidium iodide/RNase buffer (BD Biosciences, San Jose, CA, U.S.A.), according to the manufacturer’s instructions. Cell cycle analysis was carried out on an FACScalibur flow cytometer with the CellQuest software (BD Biosciences). These experiments were performed a minimum of three times.

### Microtubule regrowth assay

To assess the ability of microtubule regrowth after knockdown (KD) Cfap58 protein in astrocytes, the microtubules were completely depolymerized using 3.3 μM of nocodazole for 4 h. Next, the cells were quickly washed three times in warm proliferation medium and incubated in the medium at 37°C for 0, 30, 300 s before fixation, respectively.

### Data processing and statistical analysis

Statistical results were performed using GraphPad Prism 5. The unpaired, two-tailed *t* test with Welch’s correction was applied to determined statistical significance. Not significant (NS), *, ** and *** indicated *P*>0.05, *P*≤0.05, *P*≤0.01 and *P*≤0.001, respectively.

## Results

### Cfap58 is a novel protein that interacts with Odf2/Cenexin and is expressed in the basal body and sperm flagellum

**Figure 1:**
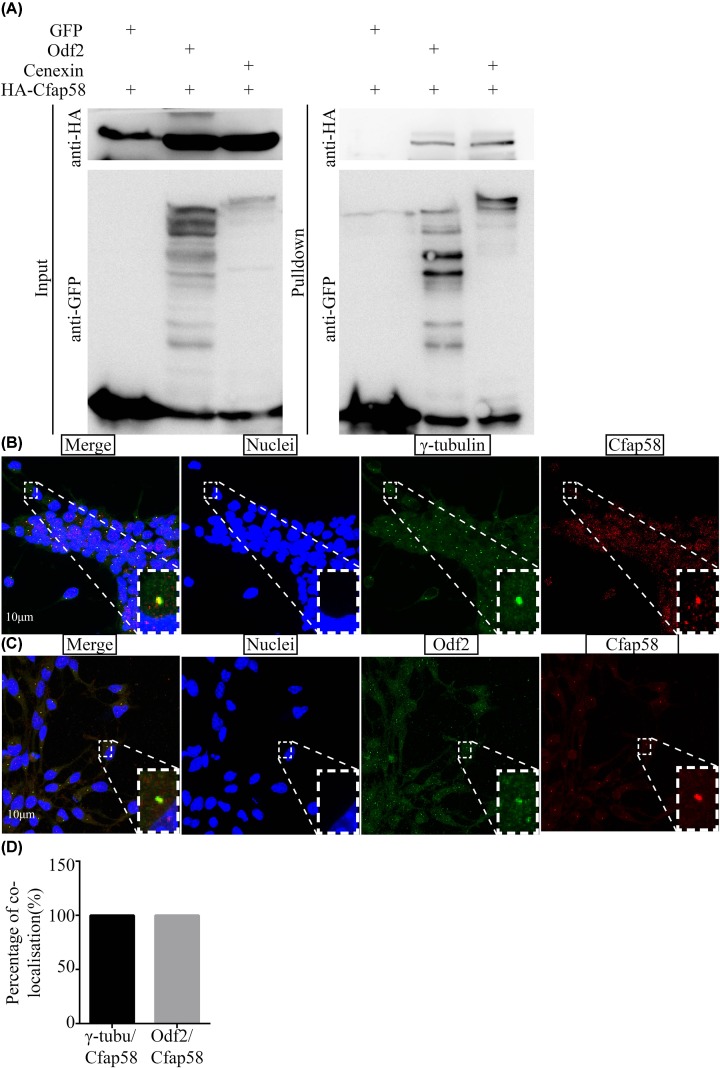
Cfap58 interacts with Odf2/Cenexin and localizes in centrosome/basal body and sperm flagellum abundantly (**A**) Western blot analysis shows the interaction between Cfap58 and Odf2/Cenexin *in vitro*. (**B**) Localization of γ-tubulin (green) and Cfap58 (red) in the centrosome in mouse astrocytes. Nuclei (blue) are stained with Hoechst34580. Scale bar = 10 μm. (**C**) Co-localization of Odf2/Cenexin (green) and Cfap58 (red) in mouse astrocytes. Nuclei (blue) are stained with Hoechst34580. Scale bar = 10 μm. (**D**) Statistical results the percentage of localization of γ-tubulin/Cfap58 and Odf2/Cfap58 for (B,C) (*n*=50 dots/group). Data are shown as the means.

**Figure 2:**
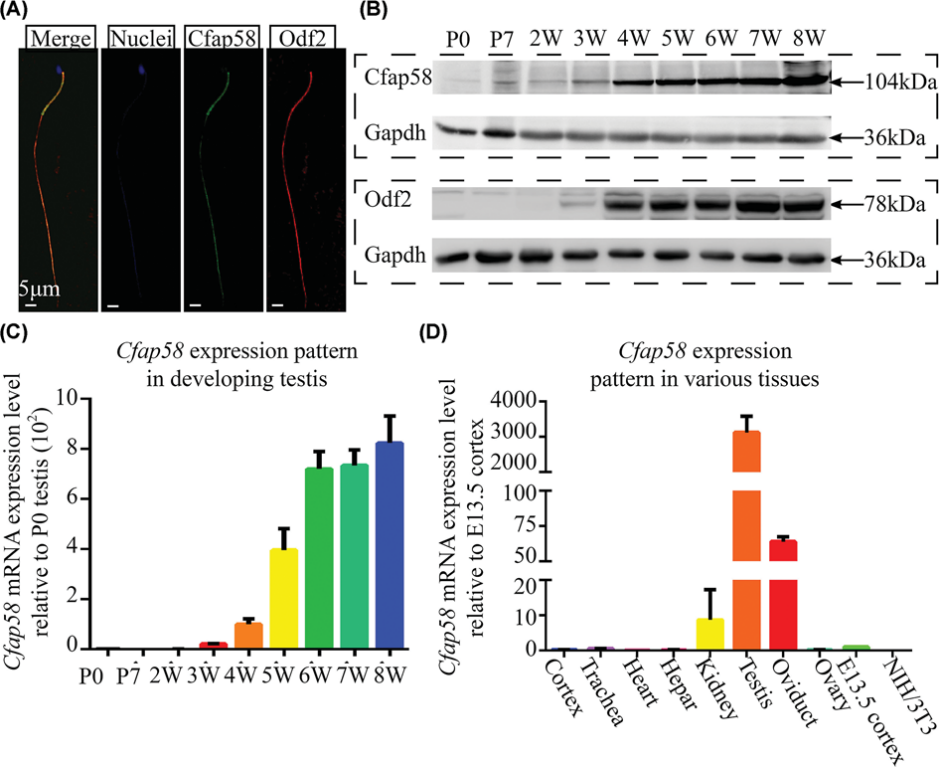
Cfap58 is a testis-enriched protein (**A**) Localization of Cfap58 (green) and Odf2 (red) in mouse sperm flagellum. Nuclei (blue) are stained with Hoechst34580. Scale bar = 5 μm. (**B**) Western blot analysis were performed that the expression patterns of Cfap58 and Odf2 in mouse developing testes. (**C,D**) qRT-PCR analysis of *Cfap58* mRNA in and developing testes (C) and mouse adult tissues (D). Mouse *Gapdh* mRNA level was an internal control. Data are shown as the means ± SEM.

To validate the interaction between Odf2 and Cfap58, co-immunoprecipitation experiments in HEK293T cells were performed as described in the ‘Materials and methods’ section. Actually, both testis-enriched isoform Odf2 and somatic cells-enriched isoform Cenexin bound with Cfap58 proteins *in vitro* (Figure 1A). To confirm the Cfap58 interaction with Odf2/Cenexin in somatic cells and sperms, immunofluorescence experiments were conducted. In neural progenitor cells, endogenous Cfap58 signals overlapped with signals of the centrosome marker, γ-tubulin (Figure 1B). And the signals of endogenous Cfap58 proteins in astrocytes partially overlapped Odf2/Cenexin signals (Figure 1C). Meanwhile, the signal of Cfap58 was mainly localized in midpiece and merged with Odf2 signals in sperms (Figure 2A). Furthermore, we examined the expression pattern of Cfap58 in developing testes and different adult tissues using Western blotting and qPCR, respectively. The expression level of Cfap58 proteins was increased during testicular development (Figure 2B,C), which was similar to the expression pattern of Odf2 in testes. qPCR with specific primers showed that Cfap58 mRNA was abundantly expressed in adult testis and detectable in ciliated cells and tissues such as neural progenitor cells and oviducts (Figure 2D). These results showed that Cfap58 interacted with Odf2 and Cenexin in different cell types and exhibited a similar expression pattern of Odf2/Cenexin in mouse cells and tissues.

### Down-regulation of Cfap58 expression does not alter the cell cycle progression and microtubule organization in astrocytes

**Figure 3:**
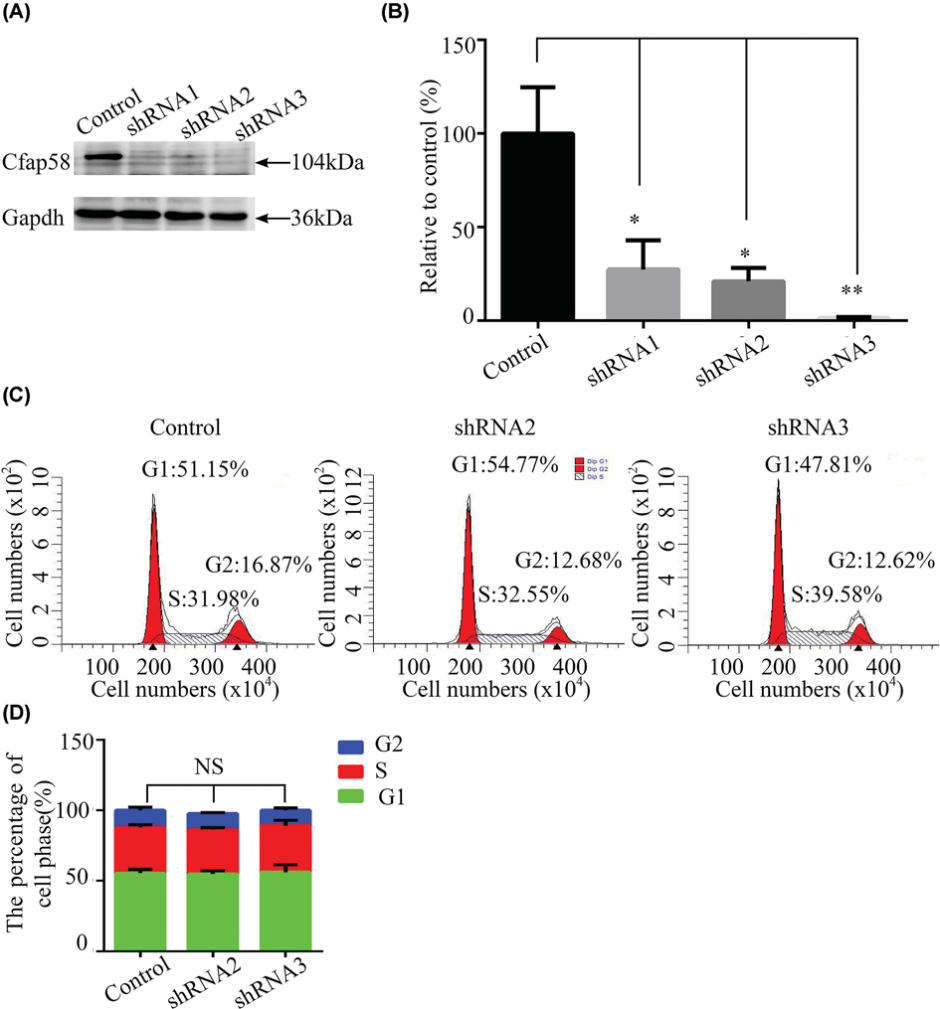
No significant alteration in cell growth and cell cycle was observed after down-regulation of Cfap58 in astrocytes (**A**) Western blot analysis exhibits validation of shRNA vectors targeted to mouse HA-Cfap58 in HEK293T cells. (**B**) Statistical result shows the silence efficiency of Cfap58 shRNA vectors. (**C,D**) Representative FACS results (C) and statistical results (D) show no significant difference in the proportion of G_0_/G_1_, G_2_/M and S phase between negative control and Cfap58 knockdown groups. Data are shown as the mean ± SEM. NS, *P*>0.05; *, *P*≤0.05; **, *P*≤0.01.

**Figure 4:**
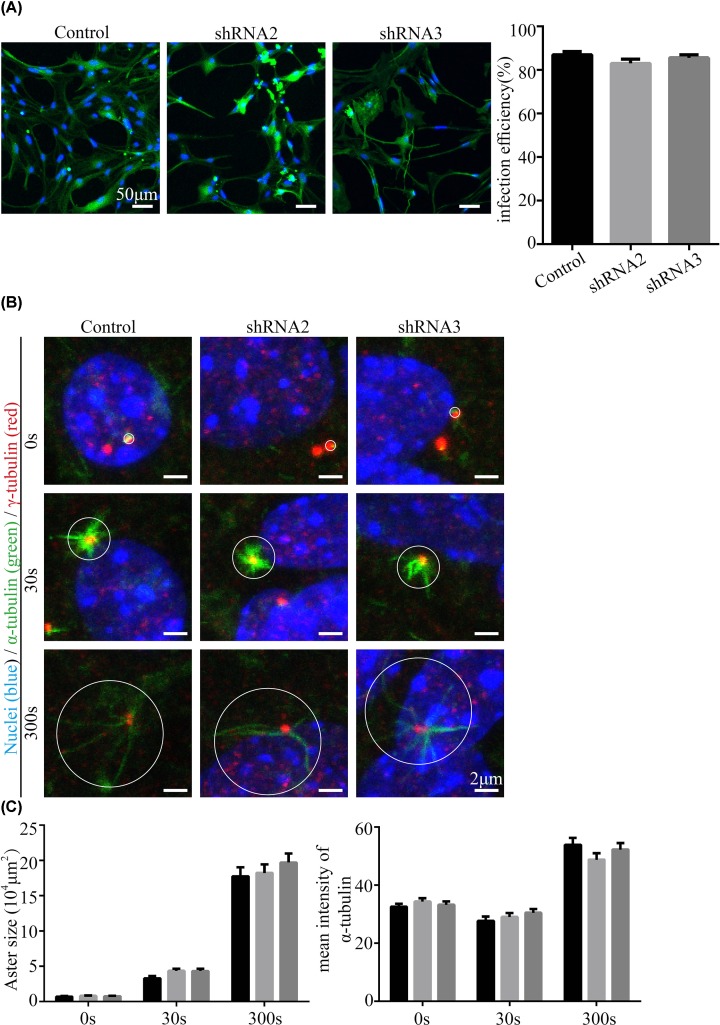
MTOC activity of the centriole does not change after the down-regulation of Cfap58 expression in astrocytes (**A**) Immunostaining data (left panel) and statistical results (right panel) show the infection efficiency of control and *Cfap58* shRNAs virus. (**B**) Centrioles stained by γ-tubulin antibody (red) show regrowth of microtubules stained by α-tubulin antibody (green) in time course. Nuclei (blue) are stained with Hoechst34580. Scale bar = 2 μm. (**C**) Statistical results show the areas and mean α-tubulin intensities of asters in (B) after nocodazole treatment in the scramble, Cfap58 shRNA2 and shRNA3 groups. (*n*≥50 asters, the white cycles indicated asters and measurements). Data are shown as the means ± SEM. NS, *P*>0.05

We next tested the effect of Cfap58 depletion by RNAi in centriolar functions. First, we designed and constructed shRNA vectors against mouse Cfap58. And then, we validated the silencing efficiency of Cfap58 shRNA vectors by transfected HEK293T cells with HA-tagged mouse Cfap58 combined with shRNA vectors, respectively. Western blotting analyses examined the expression levels of Cfap58 at day 3 post-transfection (Figure 3A,B). The most effective RNAi sequences, termed Cfap58 sh2 and sh3, were packaged into lentivirus for subsequent experiments. The infection efficiency was approximately 95% at 10 MOI (Figure 4A).

The astrocytes were dissociated from the P7 mouse midbrain and cultured for the cell cycle, microtubule regrowth and ciliogenesis analyses. RNAi knockdown efficiency in the mouse astrocyte experiments were validated again (Supplementary Figure S1). After 3 days of infection in the astrocytes, a depletion of Cfap58 cells appeared to grow normally, as observed using phase contrast microscopy (data not shown). Additionally, the cell cycle progression was not altered in Cfap58 knockdown cells (Cfap58 sh2 and sh3) according to FACS analysis (Figure 3C,D).

We then tested the MTOC activity of the centrioles in the Cfap58 sh2 and sh3 cells after nocodazole depolymerization. By analyzing the size and microtubule density of rebuilt aster-like structures, there were no significant changes observed in the Cfap58 knockdown cells compared with that in the control group (Figure 4B,C).

### Primary cilium assembly perturbation after reduced expression level of Cfap58 in astrocytes

**Figure 5:**
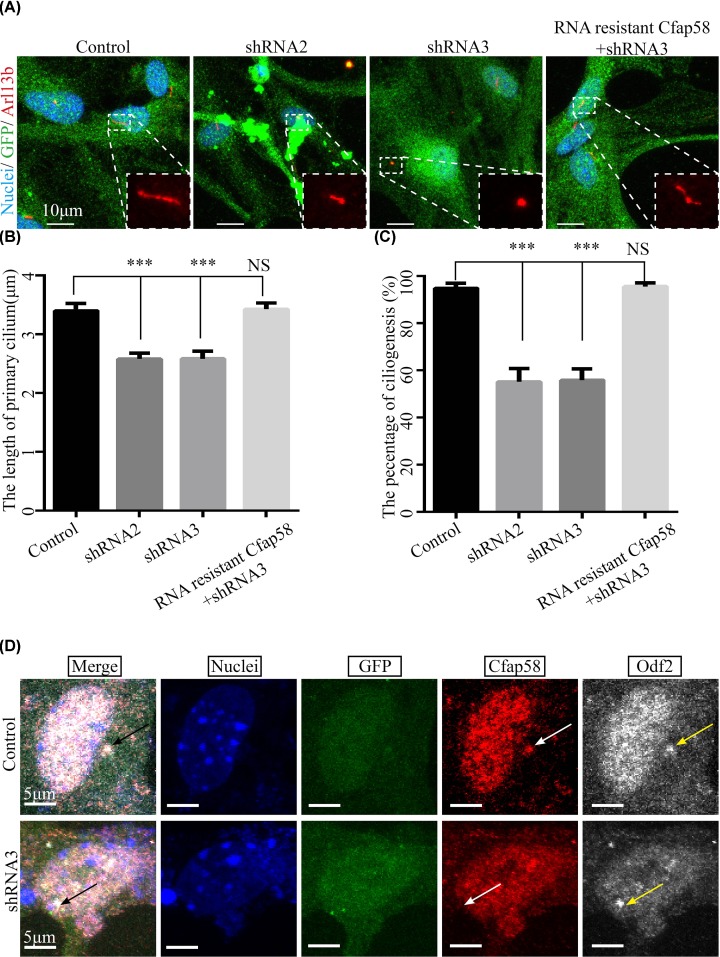
Silencing Cfap58 in astrocytes causes ciliogenesis deficiency without the mislocalization of Odf2/Cenexin (**A**) Immunostaining analysis shows that the number and length of cilia are reduced in *Cfap58* KD cells, and the RNAi-resistant *Cfap58* + shRNA3 group compared with the control group. Cells were stained with Arl13b antibody (red), GFP antibody (green) or Hoechst34580 indicating the primary cilia marker, infected cells marker and nuclei, respectively. Scale bar = 5 μm. (**B**) Statistical results show the percentages of ciliated cells in control, *Cfap58* shRNA2, shRNA3 and rescue groups. (**C**) Statistical results show the length of cilia in the scramble, *Cfap58* KD and rescue groups. (**D**) Triple immunostaining with Odf2/Cenexin (white) antibody and Cfap58 (red) antibody in control cells and *Cfap58* KD cells shows that the localization of Odf2/Cenexin is not altered in astrocytes. GFP (green) indicates infected cells. *n*=25 and 30 cells for control and *Cfap58* KD cells. Scale bar = 5 μm. Data are shown as the means ± SEM. NS, *P*>0.05; ***, *P*≤0.001.

Previous studies have reported that astrocytes process primary cilium at the G_1_/G_0_ phase [32]. Thus, we starved the astrocytes after depletion of Cfap58 to examine whether the growth of primary cilium was disrupted. As shown in Figure 5A,C, the number of ciliated cells was significantly reduced in the Cfap58 KD groups compared with that in the scramble group. Furthermore, the length of the cilia in loss of Cfap58 cells was slightly decreased (Figure 5B). The astrocytes were co-transduced with a lentiviral vector expressing the RNAi-resistant Cfap58 ORF and Cfap58 shRNA3 and showed that enforced RNAi-resistant Cfap58 expression rescued the ciliogenesis deficiency after depletion of endogenous Cfap58 (Figure 5A–C). This result supported the specificity of the RNAi-induced phenotype. Interestingly, we found the signals of Odf2/Cenexin were not apparently affected by the reduction in Cfap58 in basal body (Figure 5D). These findings indicated that Cfap58 was recruited into mother centriole by Odf2/Cenexin and indispensable for the generation of primary cilia without affecting the cell cycle progression and MTOC activity.

### Suppression of Notch signaling enhances primary ciliation and Cfap58 expression in astrocytes

**Figure 6:**
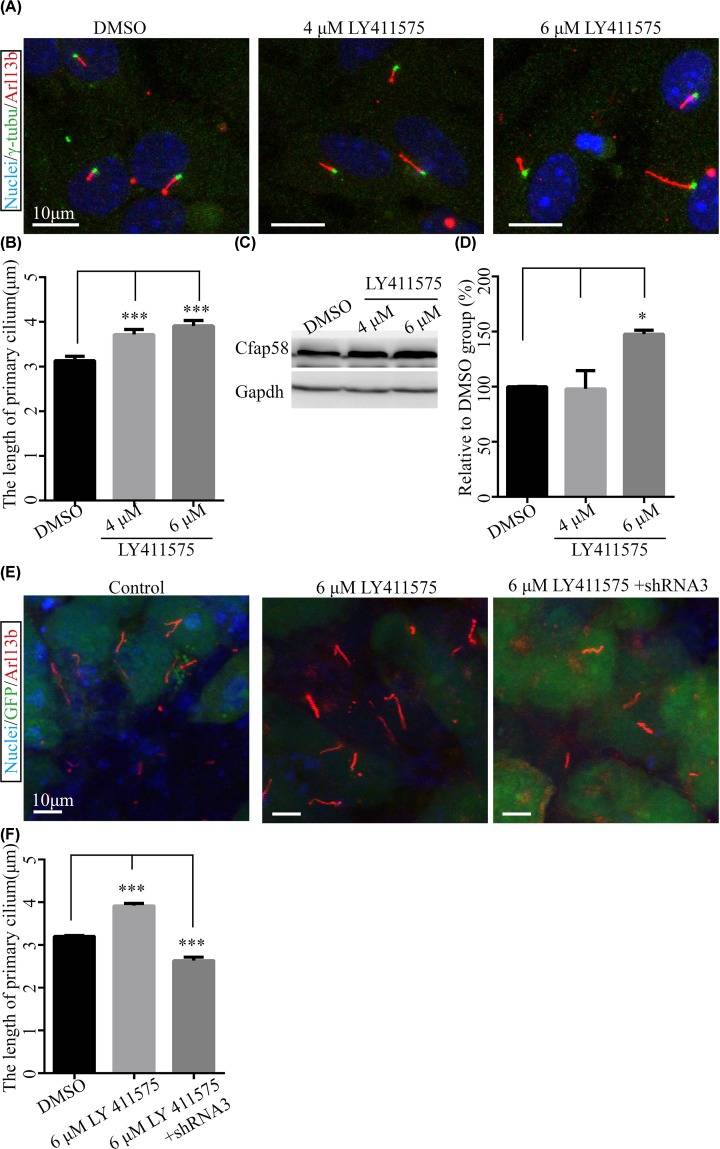
Inhibition of Notch signaling activity facilitates ciliogenesis by increasing Cfap58 protein expression in astrocytes (**A**) Representative immunofluorescent images of mouse astrocytes cultured for 3 days with 0, 4 or 6 μM LY411575 stained with Hocesht34580 (blue), γ-tubulin (green) and Arl13b antibody (red) as nuclei, basal body and primary cilia, respectively. Scale bar = 10 μm. (**B**) Statistical results show a significant increase in the length of cilia after inactivation of Notch signaling in astrocytes. (**C**) Western blot analysis shows up-regulation of Cfap58 in astrocytes treated with LY411575 in a dose-dependent manner for 3 days. The Gapdh protein level was as a loading control. (**D**) Histogram indicates the statistical results of Cfap58 expression level (three times). (**E**) Representative immunofluorescent images of mouse astrocytes treated with LY411575 for a day post-infection 3 days with the control and Cfap58 shRNA3 lentivirus. The nuclei stained with Hoechst34580 (blue), GFP (infected cells, green) and Arl13b (primary cilia). (**F**) Statistical results exhibit the length of cilia for (E). Data are shown as the means ± SEM. *, *P*≤0.05, ***, *P*≤0.001.

The functions of Notch signaling are extensively studied in various research fields [33]. The agonist and antagonist of Notch signaling are widely applied in scientific studies and clinical studies. It has been reported that primary cilium in the skin is required for Notch signaling conduction, meanwhile, the activity of Notch signaling tunes the Shh signaling activity which is dependent on the function of primary cilium [12,34,35]. Furthermore, blocking Notch signaling activity promotes the differentiation of ciliated cells in airway epithelium [36]. Thus, we wondered whether the activity of Notch signaling was involved in the ciliogenesis in astrocytes. Unexpectedly, inhibition of Notch activity not only enhanced the length of cilia, but also elevated the expression level of the Cfap58 protein (Figure 6A–D). Moreover, the effect of Notch signaling inactivation on ciliogenesis could be blocked by the reduction in Cfap58 (Figure 6E,F). However, only overexpression of Cfap58 did not increase the length of the cilia (Supplementary Figure S2). These data implied that Cfap58 is necessary but not sufficient for the regulation of primary cilia, and participates in Notch signaling-related ciliation.

### Administration of a Notch signaling inhibitor in mice facilitates the expression level of Cfap58 and the length of the sperm midpiece

**Figure 7:**
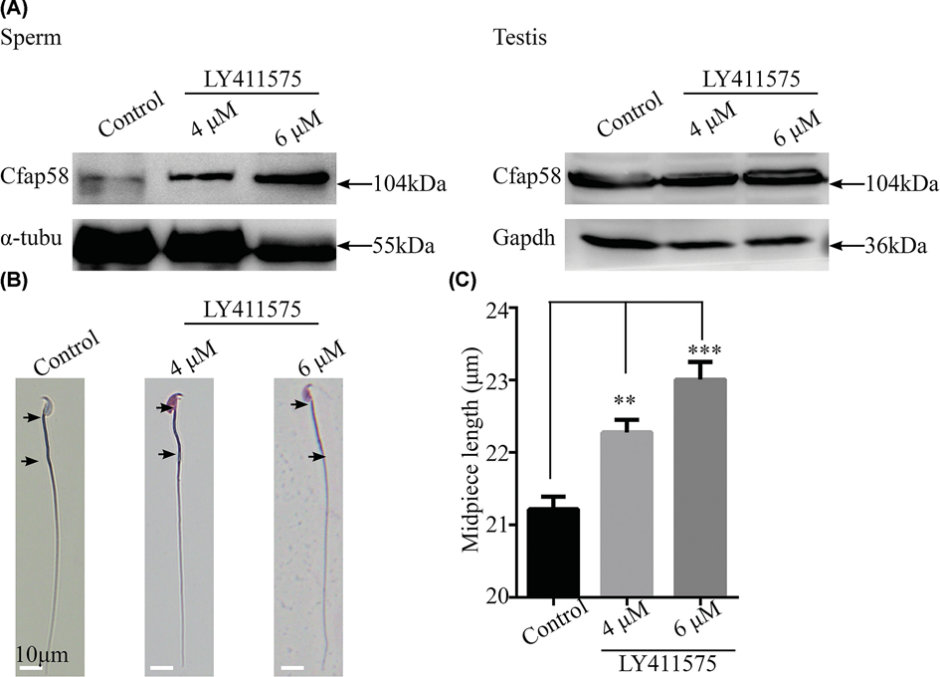
LY411575 enhances Cfap58 expression and the length of the midpiece in mouse sperm *in vivo* (**A**) Cfap58 expression is increased in mouse sperms but not adult testis. (**B**) Representative images of sperms stained by Giemsa dye to highlight the length of sperm flagellum. Black arrows indicate the region of the midpiece. (**C**) The histogram shows that the length of the midpiece is increased after LY411575 treatment for 35 days. Data are shown as the means ± SEM. **, *P*≤0.01; ***, *P*≤0.001

Next, we wondered whether the effects of a Notch signaling inhibitor existed in sperm flagella *in vivo*. To achieve this aim, we administrated the drug to mice in their drinking water in a dose-dependent manner. And then, the sperms and testes were isolated and analyzed by Western blotting and Giemsa staining. The Western blot results showed that the expression levels of Cfap58 were increased in the sperms but not testes from mice treated with LY411575 at 4 and 6 mM for 35 days (Figure 7A). Meanwhile, the length of the sperm midpiece was significantly elongated in the drug treatment groups (Figure 7B,C). These results suggested that the inhibition of Notch signaling facilitated the midpiece length by regulating the expression of Cfap58.

### Cfap58 regulates ciliogenesis independent of Aurora A activation and Nek2

Finally, we wondered whether several key components, such as Aurora A and Nek2, were involved in regulating cilia disassembly after depletion of Cfap58 [37,38]. However, we did not observe any significant changes in phosphorylated Aurora A, total Aurora A and Nek2 after down-regulation of Cfap58 (Supplementary Figure S3). The results reflect the extremely complex mechanisms underlying ciliogenesis.

## Discussion

Here, we present evidence that a testis-enriched protein, Cfap58, interacts with a vital mother centriole protein—Odf2/Cenexin and is required for the normal ciliary and flagellar architecture upon Notch signaling in cultured astrocytes and mouse sperms. However, several questions have logically arisen from the above results and should be discussed.

Cfap58 belongs to the flagella-associated proteins (FAPs) family, which is expressed in many ciliated organisms [39]. To date, only some proteins in the family have been linked to the functions of cilia and flagella. For example, the loss of function *Cfap43, Cfap44* or *Cfap65* in humans have led to flagellar abnormalities [40–42]. The mutants in *Cfap59* (also known as *Ccdc39*) resulted in aberrant inner dynein arms and the dynein regulatory complex were identified in primary ciliary dyskinesia (PCD) patients [40]. Concomitantly, oligoasthenospermia and mild defects in sperm flagella were presented in some cases of PCD [40]. Our study clearly shows the evidence that Cfap58 is required for the ciliogenesis in cultured cells and elongation of sperm flagella. Although *Cfap58* knockout mouse model was not generated in the present study, it strongly supports the involvement of Cfap58 in spermatogenesis and ciliogenesis *in vivo*, as supported by previous and current results. In addition, it is worth noting that Cfap58 seems to be required for primary cilia formation. It is necessary to further investigate about the function of Cfap58 in a tissue-specific manner via analyzing the phenotypes in *Cfap58* knockout mice.

Previous studies have demonstrated that Odf2/Cenexin are required for the primary cilia development, basal feet formation and ODF structure architecture both *in vivo* and *in vitro* as well as the recruitment of appendage components into the mother centrioles [43,44]. To date, there are few proteins interacting with Odf2/Cenexin proteins have been identified in cells and sperms. For example, the proteins named Chibby and Trichoplein were validated as partners of Odf2/Cenexin in cells [45–47]. Depletion of Chibby led to failure of primary cilium assembly and enlargement of distal tips of motile cilia in mouse bronchial epithelium. The interaction between Chibby and Cenexin would alleviate the antagonist effect of Chibby in canonical Wnt pathway through decreasing the amount of β-catenin–Chibby complex. RNAi-mediated Trichoplein depletion impaired the MT anchoring and recruitment of ninein to centrosome. Interestingly, the depletion of Cfap58, Chibby or Trichoplein does not abolish the localization of Odf2 at the mother centriole. The immunocytochemical observations about Cfap58 and Trichoplein revealed that they did not completely overlap with Odf2 in the centrosome. This evidence suggests that Odf2/Cenexin might mediate different complex formation with different partners to implement specific cellular functions. On the other hand, Aurora A and Nek2 are considered to be two independent components in primary cilium regulation [37,38]. However, we found that Cfap58 may regulate ciliation independent of Aurora A and Nek2. Actually, Cfap58 is not associated with cell cycle progression, whereas both Aurora A and Nek2 play critical roles in mitosis and spindle formation [48,49]. It was recently reported that Fbxo41, a novel Skp1/Cullin1/F-box (SCF) E3 ligase complex subunit, regulates cilia disassembly through different mechanisms in mitotic cells and post-mitotic cells [50]. Regardless, Cfap58 is a novel Odf2/Cenexin interactional partner. The precise functions in cilia-mediated signaling and other cellular events should be further investigated in the future.

The Notch signaling pathway is a critical pathway that has been extensively studied. Previously, it has been reported that Notch signaling tunes Shh activity in the neural progenitor by regulating trafficking Patched1 and Smoothened proteins in primary cilium [51]. Sustained activation of Notch signaling repressed the multiciliated differentiation in choroid plexus tumor cells and bronchial-ciliated cell differentiation [34,36]. Alternatively, primary cilia mediate the conduction of Notch signaling. The loss of primary cilia in skin diminishes Notch signaling [12]. Importantly, we found that the expression of Cfap58 and the length of primary cilia and flagella were enhanced by inhibition of Notch signaling. Our results offer new insights into the relationship between Notch signaling and primary cilium. Furthermore, the length of sperm midpiece is considered a critical determining factor affecting sperm velocity and fertility success [52,53]. Thus, understanding the interaction among Notch signaling, ciliogenesis and flagellar architecture *in vivo* is a valuable research arena to fully explore the therapeutic potential of Notch inhibitors in tumors, ciliopathies and male infertility.

## Supplementary Material

Supplementary Figures S1-S3

Tables S1-S3

## Abbreviations

BBS: Bardet–Biedl syndrome
CASA: computer-assisted sperm analysis software
Cfap58: cilia and flagella associated protein 58
EGF: Epidermal Growth Factor
FAP: Flagella-associated proteins
FBS: Fetal Bovine Serum
FGF2: Fibroblast growth factor 2
IFT: intraflagellar transport
KD: knockdown
MOI: multiplicity of infection
MTOC: microtubule organization center
NS: not significant
ODF: outer dense fiber
Odf2: outer dense fiber 2
PCD: Primary ciliary dyskinesia
PFA: Paraformaldehyde
PKD: Polycystic kidney disease
qPCR: Real time Quantitative PCR
Shh: Sonic Hedgehog
SPF: specific-pathogen-free

## References

[1] Gerdes J.M., Davis E.E. and Katsanis N. (2009) The vertebrate primary cilium in development, homeostasis, and disease. Cell 137, 32–45 10.1016/j.cell.2009.03.023 19345185 PMC3016012

[2] Lindemann C.B. and Lesich K.A. (2016) Functional anatomy of the mammalian sperm flagellum. Cytoskeleton (Hoboken) 73, 652–669 10.1002/cm.21338 27712041

[3] Lee M.S., Hwang K.S., Oh H.W. et al. (2015) IFT46 plays an essential role in cilia development. Dev. Biol. 400, 248–257 10.1016/j.ydbio.2015.02.009 25722189 PMC4385464

[4] Zhao H., Zhu L., Zhu Y. et al. (2013) The Cep63 paralogue Deup1 enables massive de novo centriole biogenesis for vertebrate multiciliogenesis. Nat. Cell Biol. 15, 1434–1444 10.1038/ncb2880 24240477

[5] Wu J., Bao J., Kim M. et al. (2014) Two miRNA clusters, miR-34b/c and miR-449, are essential for normal brain development, motile ciliogenesis, and spermatogenesis. Proc. Natl. Acad. Sci. U.S.A. 111, E2851–E2857 10.1073/pnas.1407777111 24982181 PMC4104921

[6] Nemajerova A., Kramer D., Siller S.S. et al. (2016) TAp73 is a central transcriptional regulator of airway multiciliogenesis. Genes Dev. 30, 1300–1312 10.1101/gad.279836.116 27257214 PMC4911929

[7] Abdelhamed Z., Vuong S.M., Hill L. et al. (2018) A mutation in Ccdc39 causes neonatal hydrocephalus with abnormal motile cilia development in mice. Development 145, pii: dev154500 10.1242/dev.154500 29317443 PMC5825874

[8] Faubel R., Westendorf C., Bodenschatz E. and Eichele G. (2016) Cilia-based flow network in the brain ventricles. Science 353, 176–178 10.1126/science.aae0450 27387952

[9] Suarez S.S. (1987) Sperm transport and motility in the mouse oviduct: observations *in situ*. Biol. Reprod. 36, 203–210 10.1095/biolreprod36.1.203 3567275

[10] Zimmerman K. and Yoder B.K. (2015) SnapShot: sensing and signaling by cilia. Cell 161, 692e1–692e1 10.1016/j.cell.2015.04.015 25910215 PMC4757474

[11] Falcon-Urrutia P., Carrasco C.M., Lois P., Palma V. and Roth A.D. (2015) Shh signaling through the primary cilium modulates rat oligodendrocyte differentiation. PLoS ONE 10, e0133567 10.1371/journal.pone.0133567 26218245 PMC4517900

[12] Ezratty E.J., Stokes N., Chai S. et al. (2011) A role for the primary cilium in Notch signaling and epidermal differentiation during skin development. Cell 145, 1129–1141 10.1016/j.cell.2011.05.030 21703454 PMC3135909

[13] Lancaster M.A., Louie C.M., Silhavy J.L. et al. (2009) Impaired Wnt-beta-catenin signaling disrupts adult renal homeostasis and leads to cystic kidney ciliopathy. Nat. Med. 15, 1046–1054 10.1038/nm.2010 19718039 PMC2895985

[14] Lancaster M.A. and Gleeson J.G. (2009) The primary cilium as a cellular signaling center: lessons from disease. Curr. Opin. Genet. Dev. 19, 220–229 10.1016/j.gde.2009.04.008 19477114 PMC2953615

[15] Wheeler R.J., Gluenz E. and Gull K. (2015) Basal body multipotency and axonemal remodelling are two pathways to a 9+0 flagellum. Nat. Commun. 6, 8964 10.1038/ncomms9964 26667778 PMC4682162

[16] Zhao W., Li Z., Ping P. et al. (2018) Outer dense fibers stabilize the axoneme to maintain sperm motility. J. Cell. Mol. Med. 22, 1755–1768 10.1111/jcmm.13457 29168316 PMC5824370

[17] Haidl G., Becker A. and Henkel R. (1991) Poor development of outer dense fibers as a major cause of tail abnormalities in the spermatozoa of asthenoteratozoospermic men. Hum. Reprod. 6, 1431–1438 10.1093/oxfordjournals.humrep.a137283 1770140

[18] Konno A., Shiba K., Cai C. and Inaba K. (2015) Branchial cilia and sperm flagella recruit distinct axonemal components. PLoS ONE 10, e0126005 10.1371/journal.pone.0126005 25962172 PMC4427456

[19] Kierszenbaum A.L., Rivkin E., Tres L.L. et al. (2011) GMAP210 and IFT88 are present in the spermatid golgi apparatus and participate in the development of the acrosome-acroplaxome complex, head-tail coupling apparatus and tail. Dev. Dyn. 240, 723–736 10.1002/dvdy.22563 21337470 PMC4175411

[20] Liu P. and Lechtreck K.F. (2018) The Bardet-Biedl syndrome protein complex is an adapter expanding the cargo range of intraflagellar transport trains for ciliary export. Proc. Natl. Acad. Sci. U.S.A. 115, E934–E943 10.1073/pnas.1713226115 29339469 PMC5798339

[21] Pedersen L B. and Christensen S.T. (2012) Regulating intraflagellar transport. Nat. Cell Biol. 14, 904–906 10.1038/ncb2569 22945257

[22] Kim J., Lee J.E., Heynen-Genel S. et al. (2010) Functional genomic screen for modulators of ciliogenesis and cilium length. Nature 464, 1048–1051 10.1038/nature08895 20393563 PMC2929961

[23] Nakagawa Y., Yamane Y., Okanoue T., Tsukita S. and Tsukita S. (2001) Outer dense fiber 2 is a widespread centrosome scaffold component preferentially associated with mother centrioles: its identification from isolated centrosomes. Mol. Biol. Cell 12, 1687–1697 10.1091/mbc.12.6.1687 11408577 PMC37333

[24] Flickinger C.J., Rao J., Bush L.A. et al. (2001) Outer dense fiber proteins are dominant postobstruction autoantigens in adult Lewis rats. Biol. Reprod. 64, 1451–1459 10.1095/biolreprod64.5.1451 11319151

[25] Ishikawa H., Kubo A., Tsukita S. and Tsukita S. (2005) Odf2-deficient mother centrioles lack distal/subdistal appendages and the ability to generate primary cilia. Nat. Cell Biol. 7, 517–524 10.1038/ncb1251 15852003

[26] McCarthy K D. and de Vellis J. (1980) Preparation of separate astroglial and oligodendroglial cell cultures from rat cerebral tissue. J. Cell Biol. 85, 890–902 10.1083/jcb.85.3.890 6248568 PMC2111442

[27] Stubblefield K., Chean J., Nguyen T., Chen C.J. and Shively J.E. (2017) The adaptor SASH1 acts through NOTCH1 and its inhibitor DLK1 in a 3D model of lumenogenesis involving CEACAM1. Exp. Cell Res. 359, 384–393 10.1016/j.yexcr.2017.08.022 28823832 PMC8994489

[28] Liu X., Gu W. and Li X. (2013) HLA-G regulates the invasive properties of JEG-3 choriocarcinoma cells by controlling STAT3 activation. Placenta 34, 1044–1052 10.1016/j.placenta.2013.07.070 24054889

[29] Liu X., Zhao W., Liu H. et al. (2016) Developmental and functional brain impairment in offspring from preeclampsia-like rats. Mol. Neurobiol. 53, 1009–1019 10.1007/s12035-014-9060-7 25575681 PMC4752589

[30] Cao J., Shen Y., Zhu L. et al. (2012) miR-129-3p controls cilia assembly by regulating CP110 and actin dynamics. Nat. Cell Biol. 14, 697–706 10.1038/ncb2512 22684256

[31] Warmflash A., Sorre B., Etoc F., Siggia E.D. and Brivanlou A.H. (2014) A method to recapitulate early embryonic spatial patterning in human embryonic stem cells. Nat. Methods 11, 847–854 10.1038/nmeth.3016 24973948 PMC4341966

[32] Sterpka A., Chen X. (2018) Neuronal and astrocytic primary cilia in the mature brain. Pharmacol Res. 137, 114–121 30291873 10.1016/j.phrs.2018.10.002 PMC6410375

[33] Siebel C. and Lendahl U. (2017) Notch signaling in development, tissue homeostasis, and disease. Physiol. Rev. 97, 1235–1294 10.1152/physrev.00005.2017 28794168

[34] Li L., Grausa K.B., Wang J. et al. (2016) Sonic Hedgehog promotes proliferation of Notch-dependent monociliated choroid plexus tumour cells. Nat. Cell Biol. 18, 418–430 10.1038/ncb3327 26999738 PMC4814324

[35] Liu Y., Pathak N., Kramer-Zucker A. and Drummond I.A. (2007) Notch signaling controls the differentiation of transporting epithelia and multiciliated cells in the zebrafish pronephros. Development 134, 1111–1122 10.1242/dev.02806 17287248

[36] Gerovac B.J., Valencia M., Baumlin N. et al. (2014) Submersion and hypoxia inhibit ciliated cell differentiation in a notch-dependent manner. Am. J. Respir. Cell Mol. Biol. 51, 516–525 10.1165/rcmb.2013-0237OC 24754775 PMC4189480

[37] Pugacheva E.N., Jablonski S.A., Hartman T.R., Henske E.P. and Golemis E.A. (2007) HEF1-dependent Aurora A activation induces disassembly of the primary cilium. Cell 129, 1351–1363 10.1016/j.cell.2007.04.035 17604723 PMC2504417

[38] Kim S., Lee K., Choi J.H., Ringstad N. and Dynlacht B.D. (2015) Nek2 activation of Kif24 ensures cilium disassembly during the cell cycle. Nat. Commun. 6, 8087 10.1038/ncomms9087 26290419 PMC4545512

[39] Pazour G.J., Agrin N., Leszyk J. and Witman G.B. (2005) Proteomic analysis of a eukaryotic cilium. J. Cell Biol. 170, 103–113 10.1083/jcb.200504008 15998802 PMC2171396

[40] Merveille A.C., Davis E.E., Becker-Heck A. et al. (2011) CCDC39 is required for assembly of inner dynein arms and the dynein regulatory complex and for normal ciliary motility in humans and dogs. Nat. Genet. 43, 72–78 10.1038/ng.726 21131972 PMC3509786

[41] Firat-Karalar E.N., Sante J., Elliott S. and Stearns T. (2014) Proteomic analysis of mammalian sperm cells identifies new components of the centrosome. J. Cell Sci. 127, 4128–4133 10.1242/jcs.157008 25074808 PMC4179487

[42] Imsland F., Feng C., Boije H. et al. (2012) The Rose-comb mutation in chickens constitutes a structural rearrangement causing both altered comb morphology and defective sperm motility. PLoS Genet. 8, e1002775 10.1371/journal.pgen.1002775 22761584 PMC3386170

[43] Kunimoto K., Yamazaki Y., Nishida T. et al. (2012) Coordinated ciliary beating requires Odf2-mediated polarization of basal bodies via basal feet. Cell 148, 189–200 10.1016/j.cell.2011.10.052 22265411

[44] Salmon N.A., Reijo Pera R.A. and Xu E.Y. (2006) A gene trap knockout of the abundant sperm tail protein, outer dense fiber 2, results in preimplantation lethality. Genesis 44, 515–522 10.1002/dvg.20241 17078042 PMC3038656

[45] Siller S.S., Burke M.C., Li F.Q. and Takemaru K.I. (2015) Chibby functions to preserve normal ciliary morphology through the regulation of intraflagellar transport in airway ciliated cells. Cell Cycle 14, 3163–3172 26266958 10.1080/15384101.2015.1080396 PMC4825556

[46] Ibi M., Zou P., Inoko A. et al. (2011) Trichoplein controls microtubule anchoring at the centrosome by binding to Odf2 and ninein. J. Cell Sci. 124, 857–864 10.1242/jcs.075705 21325031

[47] Steere N., Chae V., Burke M. et al. (2012) A Wnt/beta-catenin pathway antagonist Chibby binds Cenexin at the distal end of mother centrioles and functions in primary cilia formation. PLoS ONE 7, e41077 10.1371/journal.pone.0041077 22911743 PMC3401179

[48] Hu C.M., Zhu J., Guo X.E. et al. (2015) Novel small molecules disrupting Hec1/Nek2 interaction ablate tumor progression by triggering Nek2 degradation through a death-trap mechanism. Oncogene 34, 1220–1230 10.1038/onc.2014.67 24662830 PMC4175300

[49] Plotnikova O.V., Nikonova A.S., Loskutov Y.V. et al. (2012) Calmodulin activation of Aurora-A kinase (AURKA) is required during ciliary disassembly and in mitosis. Mol. Biol. Cell 23, 2658–2670 10.1091/mbc.e11-12-1056 22621899 PMC3395655

[50] King C.R., Quadros A.R.A.A., Chazeau A. et al. (2019) Fbxo41 promotes disassembly of neuronal primary cilia. Sci. Rep. 9, 8179 10.1038/s41598-019-44589-2 31160656 PMC6546786

[51] Kong J.H., Yang L., Dessaud E. et al. (2015) Notch activity modulates the responsiveness of neural progenitors to sonic hedgehog signaling. Dev. Cell 33, 373–387 10.1016/j.devcel.2015.03.005 25936505 PMC4449290

[52] Firman R C. and Simmons L.W. (2010) Sperm midpiece length predicts sperm swimming velocity in house mice. Biol. Lett. 6, 513–516 10.1098/rsbl.2009.1027 20147311 PMC2936205

[53] Bennison C., Hemmings N., Slate J. and Birkhead T. (2015) Long sperm fertilize more eggs in a bird. Proc. Biol. Sci. 282, 20141897 10.1098/rspb.2014.1897 25621327 PMC4286041

