# Supplementary material for: The novel testicular enrichment protein Cfap58 is required for Notch-associated ciliogenesis
Source: Biosci Rep. 2020 Jan 17;40(1):BSR20192666. doi: 10.1042/BSR20192666 (PMC6970087; doi:10.1042/BSR20192666)
Supplement: Supplementary Figures S1-S3 [file BSR-2019-2666_supp.pdf]

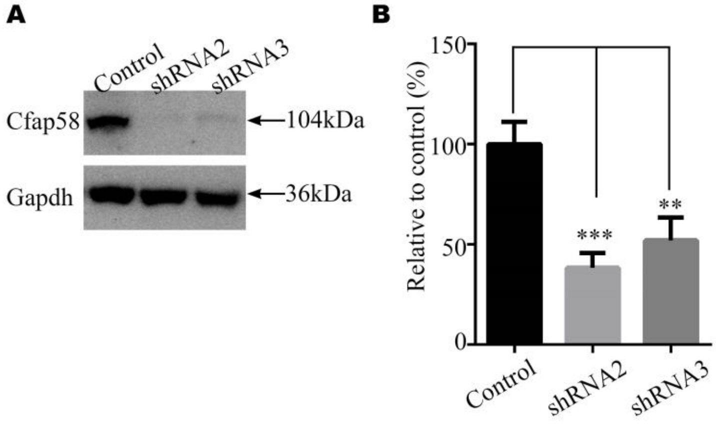
A
Control
shRNA2
shRNA3
Cfap58
104kDa
Gapdh
36kDa
B
Relative to control (%)
150
100
50
0
***
**
Control
shRNA2
shRNA3

Supplementary Figure 1:RNAi knockdown efficiency in the mouse astrocytes. (A) Western blot analysis exhibitsvalidation of shRNA vectors targeted to mouse endogenous Cfap58 in mouse astrocytes.The Gapdh level was as an internal control, (n≥4).(B) Statistical results show the silence efficiency of Cfap58 shRNA vectors.Data are shown as the means±SEM. NS, P＞0.05;*, P≤0.05; **, P≤0.01.

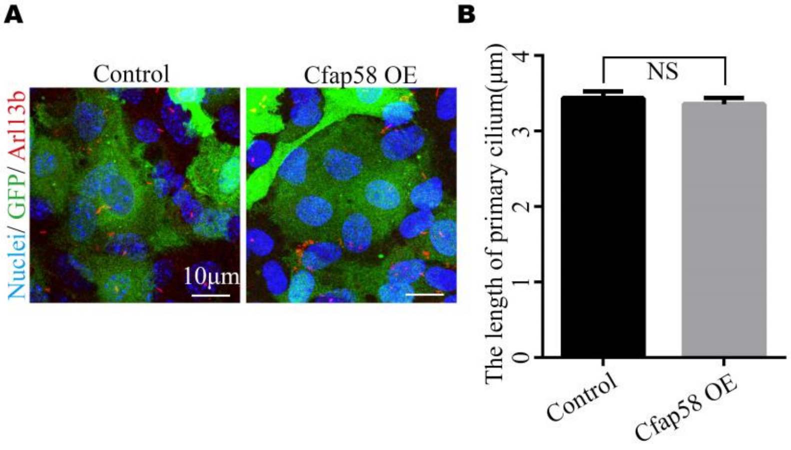
A
Control
Cfap58 OE
Nuclei/ GFP/ Arl13b
10μm
B
NS
The length of primary cilium(μm)
0
1
2
3
4
Control
Cfap58 OE

Supplementary Figure 2: Overexpression of Cfap58 in astrocytes does not enhance the length of primary cilia. (A) Representative images of the primary cilia in control and Cfap58 overexpression group. Cells are stained with Arl13b antibody (red), GFP antibody (green) or Hoechst34580 indicated as primary cilia marker, infected cells marker and nuclei, respectively. N=3 replicates. Scale bar=10μm. (B) Statistical results show the length of the primary cilia in control and Cfap58 overexpression group. Data are shown as the means ± SEM. NS, $P > 0.05$.

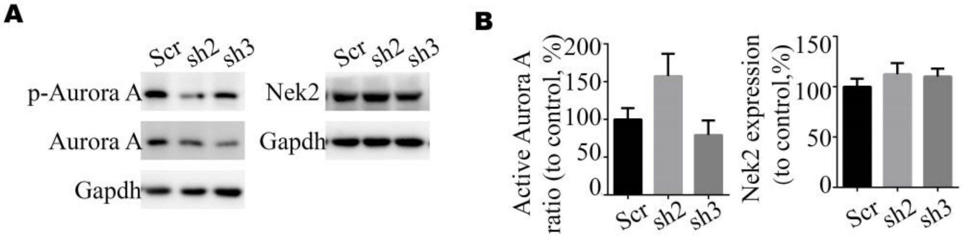
A
Scr
sh2
sh3
p-Aurora A
Aurora A
Gapdh
Nek2
Gapdh
B
Active Aurora A ratio (to control, %)
200
150
100
50
0
Scr
sh2
sh3
Nek2 expression (to control,%)
150
100
50
0
Scr
sh2
sh3

Supplementary Figure 3: Cfap58 regulates ciliogenesis independent of Aurora A activation and Nek2. (A) Representative western blotting images showing the expression of phosphorylated Aurora A, total Aurora A (right panel) and Nek2 (left panel) in control and Cfap58 KO groups.The Gapdh level was as an internal control. (B) Statistical results show the expression levelsof active Aurora A (right panel) and Nek2 (left panel), (n=3).Data are shown as the means ± SEM.
